# Development and validation of risk models to predict outcomes following in-hospital cardiac arrest attended by a hospital-based resuscitation team^☆^

**DOI:** 10.1016/j.resuscitation.2014.05.004

**Published:** 2014-08

**Authors:** David A. Harrison, Krishna Patel, Edel Nixon, Jasmeet Soar, Gary B. Smith, Carl Gwinnutt, Jerry P. Nolan, Kathryn M. Rowan, on behalf of the National Cardiac Arrest Audit

**Affiliations:** a Intensive Care National Audit & Research Centre (ICNARC), Napier House, 24 High Holborn, London WC1V 6AZ, UK; b Department of Anaesthesia and Intensive Care Medicine, Southmead Hospital, Bristol, UK; c The School of Health and Social Care, University of Bournemouth, Bournemouth, UK; d Department of Anaesthesia, Salford Royal Hospital, Salford, UK; e Department of Anaesthesia and Intensive Care Medicine, Royal United Hospital, Bath, UK

**Keywords:** Cardiopulmonary resuscitation, Heart arrest, Hospital mortality, Models, Statistical, Risk adjustment

## Abstract

**Aim:**

The National Cardiac Arrest Audit (NCAA) is the UK national clinical audit for in-hospital cardiac arrest. To make fair comparisons among health care providers, clinical indicators require case mix adjustment using a validated risk model. The aim of this study was to develop and validate risk models to predict outcomes following in-hospital cardiac arrest attended by a hospital-based resuscitation team in UK hospitals.

**Methods:**

Risk models for two outcomes—return of spontaneous circulation (ROSC) for greater than 20 min and survival to hospital discharge—were developed and validated using data for in-hospital cardiac arrests between April 2011 and March 2013. For each outcome, a full model was fitted and then simplified by testing for non-linearity, combining categories and stepwise reduction. Finally, interactions between predictors were considered. Models were assessed for discrimination, calibration and accuracy.

**Results:**

22,479 in-hospital cardiac arrests in 143 hospitals were included (14,688 development, 7791 validation). The final risk model for ROSC > 20 min included: age (non-linear), sex, prior length of stay in hospital, reason for attendance, location of arrest, presenting rhythm, and interactions between presenting rhythm and location of arrest. The model for hospital survival included the same predictors, excluding sex. Both models had acceptable performance across the range of measures, although discrimination for hospital mortality exceeded that for ROSC > 20 min (c index 0.81 versus 0.72).

**Conclusions:**

Validated risk models for ROSC > 20 min and hospital survival following in-hospital cardiac arrest have been developed. These models will strengthen comparative reporting in NCAA and support local quality improvement.

## 1 Introduction

National clinical audit has a key role to play in ensuring high quality clinical care.^1^ In 2009, the Resuscitation Council (UK) and the Intensive Care National Audit & Research Centre (ICNARC) jointly established the National Cardiac Arrest Audit (NCAA) as the UK national clinical audit of in-hospital cardiac arrest. NCAA monitors and reports on the incidence of, and outcome from, cardiac arrests attended by a hospital-based resuscitation team in order to inform practice and policy. It aims to identify deficiencies and foster improvements in the prevention, treatment and outcomes of in-hospital cardiac arrest.

In order to make fair comparisons among health care providers, clinical indicators require case mix adjustment to account for differences in the characteristics of patients that would be expected to lead to different outcomes.^2^ This is best achieved through a robust and validated statistical risk model that can estimate a predicted probability of the outcome for each individual.^3^ Although several audits and registries of in-hospital cardiac arrest have been established—most notably the American Heart Association's ‘Get With The Guidelines–Resuscitation’ (GWTG-R) registry (formerly the National Registry of Cardiopulmonary Resuscitation), ongoing since 2000^4^—the first validated risk model for outcome following in-hospital cardiac arrest was only published in 2013.^5^ Furthermore, this risk model, based on data from the United States, may not transfer well to different health care systems.^6–8^

We present the development and validation of risk models to predict outcomes following in-hospital cardiac arrests attended by a hospital-based resuscitation team in UK hospitals. These risk models will underpin comparative reporting for NCAA, to promote consistent delivery of high quality resuscitation in hospitals throughout the UK.

## 2 Methods

### 2.1 The National Cardiac Arrest Audit (NCAA)

NCAA is the national clinical audit of in-hospital cardiac arrest in UK acute hospitals. Data on demographics, risk factors and outcomes are collected for consecutive patients (adults and children) receiving cardiopulmonary resuscitation (CPR) and attended by a hospital-based resuscitation team in response to an emergency call. Standardised data are collected at the time of the cardiac arrest and from the medical record. Staff at participating hospitals enters data directly into a dedicated, secure online system. Data are validated both at the point of data entry and centrally, being checked for completeness, discrepancies and illogicalities. More detail on NCAA and the characteristics of included arrests are included in the accompanying paper.^9^

NCAA received approval from the Ethics and Confidentiality Committee of the National Information Governance Board for Health and Social Care to process limited patient identifiable data under Section 251 of the NHS Act 2006 (approval number ECC 2-06(n)/2009).

### 2.2 Inclusion and exclusion criteria

For NCAA, data are collected for all individuals (excluding neonates) receiving chest compressions and/or defibrillation and attended by a hospital-based resuscitation team (or equivalent) in response to a 2222 call (2222 is the telephone number used to summon a resuscitation team in UK hospitals).

For development of the risk models, data were extracted for all individuals meeting the scope of NCAA with a date of 2222 call between 1 April 2011 and 30 September 2012. Data for individual hospitals were included if the hospital had commenced participation in NCAA prior to April 2012 and had validated data for at least six months. Individual team visit records meeting the following criteria were considered ineligible for inclusion in the risk model: arrests that occurred pre-hospital (but were subsequently attended by a hospital-based resuscitation team and therefore met the scope of NCAA); second and subsequent visits to the same patient during the same hospital stay; and patients for whom it was identified, after starting resuscitation, that a ‘do not attempt cardiopulmonary resuscitation’ (DNACPR) decision was documented in the patient's notes. The following exclusion criteria were applied to individual team visit records: patients whose last known status was still in hospital; patients missing the outcomes of return of spontaneous circulation (ROSC) for greater than 20 min or survival to hospital discharge; patients with missing data for candidate predictors.

For validation of the risk models, data were extracted for all individuals in hospitals included in the development dataset with a date of 2222 call between 1 October 2012 and 31 March 2013 and for all individuals in hospitals that commenced participation in NCAA between April and September 2012 (and were therefore not included in the development dataset) with a date of 2222 call between 1 April 2012 and 31 March 2013. The same eligibility criteria and exclusion criteria were applied at the individual team visit level as for the development dataset.

## 3 Model development

Risk models were developed for two outcomes: ROSC greater than 20 min and survival to hospital discharge. Patients were followed up to discharge from the original hospital and any patients transferred to another acute hospital were reported as hospital survivors.

A list of candidate predictors was established from the dataset developed and collected for NCAA. A valid predictor was considered to be any variable collected prior to or at the time of the arrival of the hospital-based resuscitation team and not related to variations in the quality of care. If factors related to the quality of care were included within the risk model then the expected number of events would be adjusted to account for these factors. Consequently, a poorly performing provider would not be identified as an outlier and these discrepancies in the quality of care would not be recognised.

The full list of candidate predictors is presented in Table 1. Location of arrest was not considered to be a predictor for patients with a reason for admission to/attendance at/visit to hospital of ‘staff’ or ‘visitor’. Prior to any modelling, candidate predictors were examined for data completeness and distributions. Where categories with very few patients were identified, these were eliminated by combining with other categories. Multicollinearity between candidate predictors was assessed with variance inflation factors.

Age was modelled as a continuous, nonlinear relationship using restricted cubic splines with four degrees of freedom. All other candidate predictors were modelled as categorical variables. After examining plots of the distribution and the association with outcomes, the continuous predictor length of stay in hospital prior to 2222 call was categorised as 0 days (i.e. cardiac arrest on the same calendar day as admission to/attendance at/visit to hospital), 1 day, 2–7 days, 8–30 days and greater than 30 days.

An initial, full model for each outcome was fitted including all candidate predictors using multilevel logistic regression with random effects of hospital. The basis for using multilevel models is that, as with the majority of health outcomes, there is ‘clustering’ at the level of healthcare providers, that is, outcomes for patients within the same hospital will be, on average, more similar than outcomes for patients in different hospitals. If clustering is ignored, then the resulting model estimates will have standard errors that are too small, leading to the potential for misleading conclusions.

These models were then simplified in three stages: first, by testing for non-linearity in the relationship for age; second, by testing for differences between prespecified combinations of categories of predictors to reduce the numbers of categories; and third, by stepwise reduction of the models to reduce the number of predictors. Combining categories of predictors was conducted in such a way as to ensure the same categories were used in the models for both outcomes. This was achieved by combining categories if the difference in outcome between the categories (adjusted for all other predictors) was non-significant (*P* > 0.1) for both outcomes. The combinations considered were:

1. For prior length of stay: adjacent categories.
2. For location of arrest:
  • Adjacent categories from: emergency department (ED); emergency admissions unit (EAU); ward, obstetric area or other inpatient location; intermediate care area; coronary care unit (CCU); HDU or PHDU; and ICU, ICU/HDU or PICU.
  • Any combination of categories from: specialist treatment area; imaging department; and cardiac catheter laboratory; and, if no difference was found between any of the three previous categories, theatre and recovery.
  • Categories for clinic and non-clinical area.

Stepwise reduction was conducted separately for the two outcomes, i.e. allowing the models for ROSC greater than 20 min and hospital survival to include different combinations of predictors. At each step, the least significant predictor was removed and the reduced model assessed for discrimination (c index, equivalent to the area under the receiver operating characteristic curve)^10^, calibration (Hosmer–Lemeshow test)^11^, accuracy (Brier's score, the mean squared error between outcome and prediction,^12^ and Shapiro's R, the geometric mean of the probability assigned to the event that occurred)^13^ and model fit (Akaike Information Criterion [AIC], which penalises the log-likelihood of the model for the number of parameters included)^14^. Stepwise reduction was continued until all predictors had been removed and a model was selected to balance simplicity against model performance.

Finally, the models were further enhanced by considering interactions between predictors. The interactions considered were prespecified following presentation of an initial model with no interaction terms and requesting input from the NCAA Steering Group, representatives from hospitals participating in NCAA, and an Expert Group of clinicians, statisticians and health services researchers formed to advise on risk modelling (see ‘Acknowledgements’ section). The interactions considered were:

• age with sex;
• age with reason for attendance;
• age with presenting rhythm (non-shockable rhythms compared with shockable or unknown);
• location of arrest with presenting rhythm.

Interaction terms were added to the full model and retained if significant at *P* < 0.01. For the interaction of location of arrest with presenting rhythm, in order to reduce the potentially large number of interaction terms, combining interaction terms for similar groups of categories of both presenting rhythm (e.g. all shockable arrests, all non-shockable arrests) and location of arrest (e.g. ED and EAU, EAU and ward, CCU and cardiac catheter laboratory) was considered.

Comparisons of models (for testing linearity, combining categories, stepwise reduction and adding interactions) were performed with likelihood ratio tests.

## 4 Model validation

The resulting models were validated for discrimination, calibration and accuracy in: (1) the development dataset; (2) the full validation dataset; and (3) the validation data from hospitals that commenced participation in NCAA from April 2012 onwards and were therefore not included in the development dataset (providing true external validation in a smaller sample of hospitals). To reduce overfitting, model estimates were shrunk using the uniform (heuristic) shrinkage method of Van Houwelingen and Le Cessie.^15^

Discrimination was assessed by the c index. Calibration was assessed graphically and tested using the Hosmer–Lemeshow test for perfect calibration in ten equal sized groups by predicted probability of survival. As the Hosmer–Lemeshow test does not provide a measure of the degree of miscalibration and is very sensitive to sample size,^16^ calibration was also assessed using Cox's calibration regression, which assesses the degree of linear miscalibration by fitting a logistic regression of observed survival on the predicted log odds of survival from the risk model.^17^ Accuracy was assessed by Brier's score and Shapiro's R, and the associated approximate R-squared statistics (termed the ‘sum-of-squares’ R-squared and the ‘entropy-based’ R-squared, respectively)^18^, which are obtained by scaling each measure relative to the value achieved from a null model. Measures of model performance were calculated using the marginal predicted probabilities from the risk model, i.e. without taking into account hospital level effects, representing the predicted probability of survival for a patient with the given characteristics in an ‘average’ hospital.

The final risk models were refitted to all data (development and validation datasets combined) to maximise precision and generalisability, with shrinkage applied to reported coefficients.

All statistical analyses were performed using Stata/SE Version 10.1 (StataCorp LP, College Station, TX, USA).

## 5 Results

### 5.1 Inclusion and exclusion criteria

Between 1 April 2011 and 31 March 2013, 148 hospitals participated in NCAA (Supplemental Fig. 1). During this time there were a total of 28,987 resuscitation team visits following 2222 calls for cardiac arrest reported to NCAA. After excluding data that were still undergoing central validation (at the level of calendar months within hospitals) and hospitals with less than six months’ data, 27,998 team visits in 143 hospitals remained. After removing records that were ineligible for risk predictions and those excluded for missing data, a total of 22,479 team visits in 143 hospitals were included, 14,688 (65.3%) in the development dataset and 7791 (34.7%) in the validation dataset. Rates of missing data were very low, with only 0.1% of patients excluded from the development dataset (0.1% from the validation dataset) due to missing predictor variables and 0.1% (0.8%) due to missing outcomes, and it was therefore not necessary to consider more complex statistical methods for handling missing data. The breakdown of exclusions in the development dataset, the validation dataset, and the external validation dataset (the subset of the validation dataset from hospitals not included in the development dataset) are shown in Supplemental Table 1 and characteristics and outcomes are summarised in Table 2.

## 6 Model development

Prior to modelling, the following categories of predictors were combined to remove small categories. For reason for admission to/attendance at/visit to hospital, the categories of staff and visitor were combined. For location of arrest, the following categories were combined: ward, obstetric area and other inpatient location (noting that obstetric patients are distinguished by the separate reason for attendance field); high dependency unit (HDU) and paediatric HDU (PHDU); and intensive care unit (ICU) or ICU/HDU and paediatric ICU (PICU) (noting that paediatric patients are distinguished by age).

The initial, full model, including the main effects of all candidate predictors (Table 1), had a c index of 0.727 for ROSC greater than 20 min and 0.804 for hospital survival (Supplemental Table 2). There was no evidence of multicollinearity (all variance inflation factors < 2). Age was significantly non-linear in both models (*P* < 0.001 for ROSC greater than 20 min, *P* = 0.007 for hospital survival). After following the prespecified process for combining categories of predictors, the following categories were combined:

1. For prior length of stay: 8–30 days with greater than 30 days (*P* = 0.21 for ROSC greater than 20 min, *P* = 0.73 for hospital survival).
2. For location of arrest:
  • ward, obstetric area or other inpatient location with intermediate care area (*P* = 0.56, *P* = 0.47);
  • HDU or PHDU with ICU, ICU/HDU or PICU (*P* = 0.16, *P* = 0.40);
  • specialist treatment area with imaging department (*P* = 0.82, *P* = 0.34); and
  • clinic with non-clinical area (*P* = 0.69, *P* = 0.39).

Combining categories had a minimal effect on the measures of model performance and resulted in an improvement (decrease) in the AIC (Supplemental Table 2).

The stepwise reduction of the models is shown in Supplemental Table 2. The predictor ‘patient deteriorating (not yet arrested) at team arrival’ was removed from both models and sex was removed from the model for hospital survival. All other predictors were highly statistically significant.

After testing the prespecified interactions, a significant interaction (*P* < 0.001) was found between location of arrest and presenting rhythm in both models and so alternative categorisations for interactions between location of arrest and presenting rhythm were considered. All other interaction terms were non-significant.

**Fig. 1:**
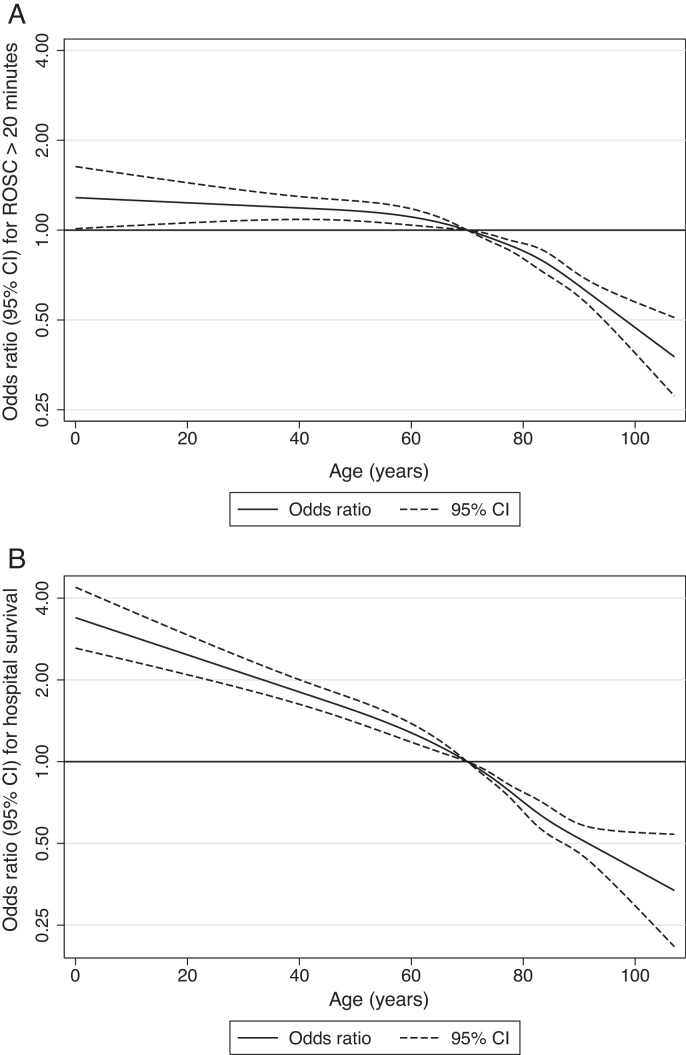
Relationship between age and: (A) return of spontaneous circulation greater than 20 min; (B) hospital survival. CI, confidence interval; ROSC, return of spontaneous circulation. Odds ratios and confidence intervals have been calculated relative to age 70 years (and therefore converge to an odds ratio of 1 at this point).

The non-linear relationships between age and outcome are illustrated in Fig. 1. For ROSC greater than 20 min, the relationship with age was flat up to around age 60, with a rapid decrease in the odds of ROSC greater than 20 min at older ages. Hospital survival decreased across the full age range, although this relationship was steeper at older ages.

## 7 Model validation

**Fig. 2:**
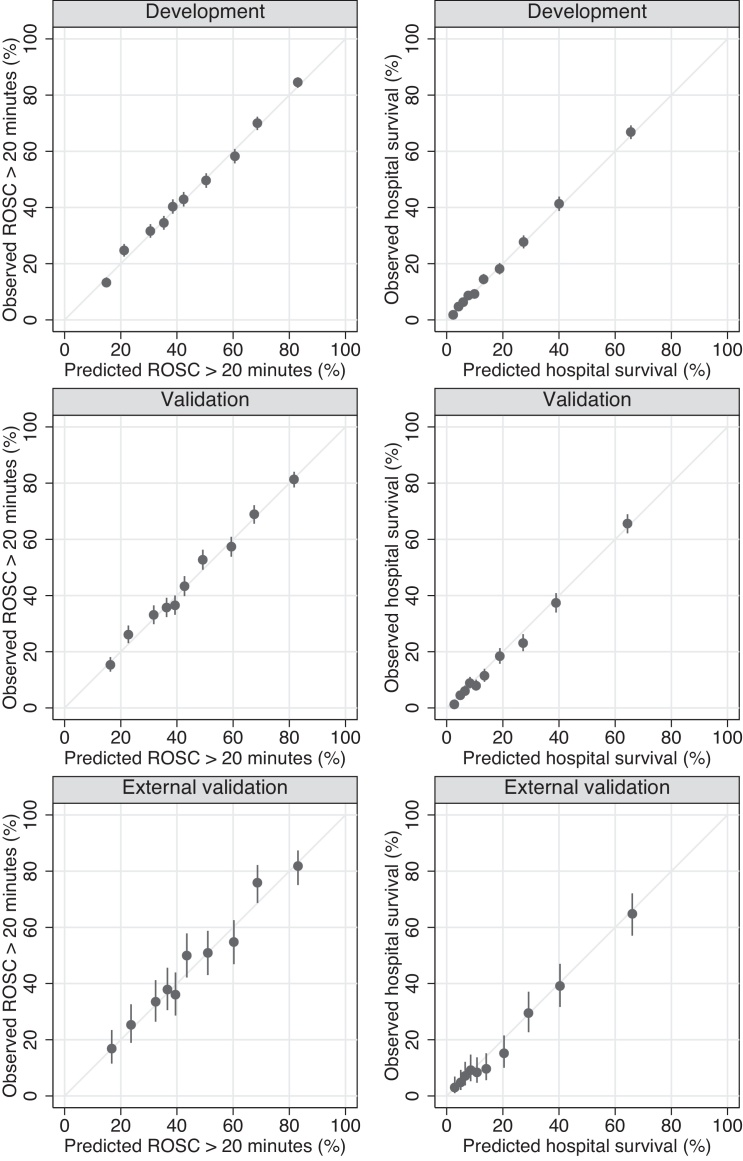
Calibration plots for return of spontaneous circulation greater than 20 min (left) and hospital survival (right) in the development, validation and external validation datasets. ROSC, return of spontaneous circulation. Observed survival (with 95% confidence interval) plotted against predicted survival in ten equal sized groups, based on models using coefficients fitted in the development dataset.

Results of the model validation, based on models fitted in the development dataset, are shown in Table 3. Discrimination and accuracy were better for hospital survival (c index ∼0.81, R-squared 0.21–0.24) than for ROSC greater than 20 min (c index ∼0.73, R-squared 0.11–0.17). Calibration was generally good, supported visually by calibration plots (Fig. 2), although there was some evidence of worse calibration for ROSC greater than 20 min in the validation dataset. Model performance was generally well preserved in the validation datasets compared with the development dataset, particularly for hospital survival. Model accuracy was also compared across age groups (Supplemental Fig. 2). Although there was some variation in outcomes (consistent with chance) in the age groups with smaller sample sizes, overall the model fit was good across all age groups. Interactions between age and other predictors were considered but were found to be unnecessary.

The final models for ROSC greater than 20 min and hospital survival, refitted to the full dataset, are shown in Supplemental Tables 3 and 4, respectively. The shrinkage factors were 0.964 and 0.970, respectively, indicating very little overfitting. A spreadsheet for automatic calculation of the predicted probability of ROSC greater than 20 min and hospital survival is provided as online supplemental material.

## 8 Discussion

Based on a relatively simple dataset, we have developed a risk model with good discrimination (c index > 0.8) for predicting survival to hospital discharge following an in-hospital cardiac arrest attended by a hospital-based resuscitation team. This model validated well in subsequent data, including external validation in data from 21 hospitals not included in the development dataset. A risk model for ROSC greater than 20 min performed less well, being potentially more sensitive to inter-hospital variation in the organisation and delivery of resuscitation practice, but still demonstrated acceptable discrimination (c index > 0.7). Although there were statistically significant departures from perfect calibration, the Hosmer–Lemeshow test is highly sensitive to sample size^16^ and graphical plots demonstrated that overall calibration was generally good in both the development and validation datasets.

The main strengths of the study are: the large, representative, high quality clinical dataset, with coverage approaching 50% of UK acute hospitals; high levels of data completeness, with only 0.3% of patients excluded due to missing data; and robust statistical modelling techniques, including using multilevel random-effects models to account for clustering of outcomes within hospitals, using restricted cubic splines to model non-linear relationships between age and outcome, and consideration of important interactions between predictors.

There are, however, some limitations. The available predictors and outcomes were limited to those recorded in the NCAA dataset, which were in turn driven by the need to ensure that data could be collected accurately in all participating hospitals on all eligible patients. Consequently, data were not available for some variables that have been found to be significant predictors of outcome in previous studies of in-hospital cardiac arrest, for example, pre-arrest comorbidities and interventions. Also, patients were followed up to discharge from the original hospital only, with any patients transferred to another hospital recorded as survivors. Data linkage with death registrations may permit this to be addressed in future by modelling survival to 30 days, 90 days or 1 year, regardless of location of death. Finally, the risk models produced predict only survival and not functional outcome. Although Cerebral Performance Category (CPC) is recorded in the NCAA dataset, we have concerns over the quality of these data due to local variations in methods of assessment and documentation.

The only existing validated risk model for in-hospital cardiac arrest (developed contemporaneously with those presented here) is from the GWTG-R registry.^5^ There are several differences between our models and the GWTG-R model for hospital survival in terms of inclusion criteria and available predictors; however, there are also many similarities. GWTG-R is a registry of all in-hospital cardiac arrests, whereas NCAA is a national clinical audit monitoring outcomes of hospital-based resuscitation teams. Consequently, while the majority of arrests in the GWTG-R registry occurred in monitored areas, in the UK many of these are managed by staff in the local unit and would not result in an emergency call to the resuscitation team and consequently would not meet the scope of NCAA. In terms of predictors included in the models, the GWTG-R model includes pre-arrest comorbidities and interventions, which are not currently available in the NCAA dataset. Other predictors included in the models were similar. The discrimination of the NCAA model for hospital survival (c index 0.811) exceeded that of the GWTG-R model (0.734) and also of a previous more complex model from the same database (0.780).^19^

The findings of our research are consistent with the recognised benefits of: (a) the patient being monitored before an arrest; (b) the arrest being witnessed; (c) staff with advanced life support skills being available in the immediate vicinity of the arrest; and (d) equipment and drugs necessary to treat the arrest being immediately available. These are all more likely to exist when the arrest occurs in a critical care unit or CCU. We found that both asystole and pulseless electrical activity (PEA) were always less likely to result in ROSC and survival to hospital discharge than ventricular fibrillation (VF). It is well recognised that for asystole and PEA the specific treatment necessary may be unclear whereas for VF the essential therapy – defibrillation – is readily available in most clinical areas of hospitals. Further, asystole may occur following VF, and is recognised to be a ‘less survivable’ rhythm. Both ROSC and survival to hospital discharge were more likely when asystole occurred on the critical care unit or CCU than the ward (odds ratios 4.82 and 5.43 for ROSC, 4.92 and 12.55 for survival, for the critical care unit and CCU, respectively). Similarly, ROSC was also more likely when VF occurred on the critical care unit or CCU than the ward (odds ratios 1.22 and 2.46, respectively). However, although survival was more likely when VF occurred on the CCU than the ward (odds ratio 3.32), this was not the case for VF occurring on a critical care unit (odds ratio 0.90), likely representing the underlying severity of illness of patients on the critical care unit.

## 9 Conclusions

We have developed and validated risk models for predicting ROSC greater than 20 min and hospital survival following in-hospital cardiac arrests attended by a hospital-based resuscitation team. These risk models are already being introduced into routine reporting for NCAA, to strengthen comparative reporting and support local quality improvement. The models will be regularly recalibrated to ensure ongoing fit and contemporaneous comparisons. Future risk modelling work for NCAA will consider linkage with death registration to model mortality following discharge from hospital and longer-term outcome, further investigation of the accuracy of functional outcome data to enable extension of the models to predict this outcome, and expanding the NCAA dataset to consider additional potentially important predictors of outcome.

## Conflicts of interest statement

All authors declare that they have no conflicts of interest.

## Role of the funding sources

This project was supported by internal funding from the Resuscitation Council (UK) and the Intensive Care National Audit & Research Centre, and by the National Institute for Health Research Health Services and Delivery Research (NIHR HS&DR) programme (project number 09/2000/65). Visit the HS&DR website for more information. The views and opinions expressed therein are those of the authors and do not necessarily reflect those of the HS&DR Programme, NIHR, NHS or the Department of Health.

The study sponsor had no involvement in the study design, in the collection, analysis and interpretation of data, in the writing of the manuscript, or in the decision to submit the manuscript for publication.

## Figures and Tables

**Table 1:** List of candidate predictors and approach to modelling each predictor.

Candidate predictors	Approach to modelling
Age	Restricted cubic splines with 4 degrees of freedom
Sex	Categorical (male; female)
LOS in hospital prior to 2222 call	Categorical (0 days; 1 day; 2–7 days; 8–30 days; >30 days)
Reason for admission to/attendance at/visit to hospital	Categorical (patient–medical; patient–trauma; patient–elective surgery; patient–emergency surgery; patient–obstetric; outpatient; staff; visitor)
Location of arrest	Categorical (ED; EAU; ward; obstetric area; intermediate care area; CCU; HDU; ICU or ICU/HDU; PHDU; PICU; specialist treatment area; imaging department; cardiac catheter laboratory; theatre and recovery; other inpatient location; clinic; non-clinical area)
Patient deteriorating (not yet arrested) at team arrival	Binary (yes; no)
Presenting/first documented rhythm	Categorical (VF; VT; shockable–unknown rhythm; asystole; PEA; bradycardia; non-shockable–unknown rhythm; unknown)

CCU, coronary care unit; EAU, emergency admissions unit; ED, emergency department; HDU, high dependency unit; ICU, intensive care unit; ICU/HDU, combined intensive care and high dependency unit; LOS, length of stay; PEA, pulseless electrical activity; PHDU, paediatric high dependency unit; PICU, paediatric intensive care unit; VF, ventricular fibrillation; VT, ventricular tachycardia.

**Table 2:** Characteristics and outcomes of in-hospital cardiac arrest patients in the development and validation datasets.

Patient characteristics	Development (*N* = 14,688)	Validation (*N* = 7791)	External validation (*N* = 1657)
Age, mean (SD)	72.6 (16.4)	72.9 (16.3)	72.8 (16.3)
Sex male, *n* (%)	8422 (57.3)	4467 (57.3)	970 (58.5)
LOS in hospital prior to 2222 call, median (IQR)	2 (0, 7)	2 (0, 7)	3 (1, 8)

Reason for admission to/attendance at/visit to hospital, *n* (%)			
Patient–medical	11,837 (80.6)	6307 (81.0)	1277 (77.1)
Patient–trauma	604 (4.1)	250 (3.2)	56 (3.4)
Patient–elective surgery	981 (6.7)	480 (6.2)	102 (6.2)
Patient–emergency surgery	1043 (7.1)	663 (8.5)	198 (11.9)
Patient–obstetric	40 (0.3)	7 (0.1)	2 (0.1)
Outpatient	149 (1.0)	69 (0.9)	18 (1.1)
Staff	10 (0.1)	1 (<0.1)	1 (0.1)
Visitor	24 (0.2)	14 (0.2)	3 (0.2)
Location of arrest, *n* (%)			
Emergency department	1655 (11.3)	702 (9.0)	41 (2.5)
Emergency admissions unit	1211 (8.2)	719 (9.2)	190 (11.5)
Ward	8242 (56.1)	4582 (58.8)	1052 (63.5)
Obstetric area	29 (0.2)	6 (0.1)	2 (0.1)
Intermediate care area	46 (0.3)	9 (0.1)	0 (0)
Coronary care unit	1390 (9.5)	668 (8.6)	140 (8.4)
HDU	259 (1.8)	128 (1.6)	19 (1.1)
ICU or ICU/HDU	680 (4.6)	348 (4.5)	61 (3.7)
Paediatric HDU	15 (0.1)	11 (0.1)	1 (0.1)
Paediatric ICU	19 (0.1)	20 (0.3)	6 (0.4)
Specialist treatment area	182 (1.2)	89 (1.1)	20 (1.2)
Imaging department	205 (1.4)	89 (1.1)	20 (1.2)
Cardiac catheter laboratory	431 (2.9)	263 (3.4)	71 (4.3)
Theatre and recovery	189 (1.3)	87 (1.1)	15 (0.9)
Other inpatient location	4 (<0.1)	5 (0.1)	2 (0.1)
Clinic	46 (0.3)	32 (0.4)	10 (0.6)
Non-clinical area	85 (0.6)	33 (0.4)	7 (0.4)
Patient deteriorating (not yet arrested) at team arrival, *n* (%)	728 (5.0)	365 (4.7)	39 (2.4)
Presenting/first documented rhythm, *n* (%)			
Ventricular fibrillation	1695 (11.5)	817 (10.5)	194 (11.7)
Ventricular tachycardia	707 (4.8)	370 (4.7)	73 (4.4)
Shockable–unknown rhythm	94 (0.6)	39 (0.5)	7 (0.4)
Asystole	3572 (24.3)	1882 (24.2)	391 (23.6)
Pulseless electrical activity	7176 (48.9)	3900 (50.1)	797 (48.1)
Bradycardia	102 (0.7)	54 (0.7)	9 (0.5)
Non-shockable–unknown rhythm	314 (2.1)	178 (2.3)	45 (2.7)
Unknown	1028 (7.0)	551 (7.1)	141 (8.5)
ROSC > 20 min, *n* (%)	6605 (45.0)	3509 (45.0)	767 (46.3)
Hospital survival, *n* (%)	2926 (19.9)	1437 (18.4)	316 (19.1)

HDU, high dependency unit; ICU, intensive care unit; IQR, interquartile range; LOS, length of stay; ROSC, return of spontaneous circulation; SD, standard deviation.

**Table 3:** Validation of risk models for return of spontaneous circulation greater than 20 min and hospital survival following in-hospital cardiac arrest attended by a hospital-based resuscitation team.

Measures of model performance^a^	Development (*N* = 14,688)	Validation (*N* = 7791)	External validation (*N* = 1657)
**ROSC** **>** **20** **min**			
c index (95% CI)	0.733 (0.725, 0.741)	0.720 (0.709, 0.732)	0.725 (0.701, 0.750)
Hosmer–Lemeshow test			
Chi-squared (*P*-value)	24.6 (0.002)	15.0 (0.13)	10.4 (0.41)
Cox calibration regression			
Intercept (95% CI)	0.021 (−0.016, 0.058)	0.015 (−0.034, 0.066)	0.038 (−0.070, 0.146)
Slope (95% CI)	1.000 (0.957, 1.043)	0.989 (0.928, 1.051)	1.003 (0.870, 1.136)
Chi-squared (*P*-value)	1.3 (0.52)	0.6 (0.73)	0.5 (0.78)
Brier's score	0.206	0.211	0.210
Sum-of-squares R-squared	0.168	0.150	0.156
Shapiro's R	0.550	0.544	0.545
Entropy-based R-squared	0.131	0.115	0.120

**Hospital survival**			
c index (95% CI)	0.811 (0.802, 0.820)	0.811 (0.799, 0.824)	0.804 (0.776, 0.832)
Hosmer–Lemeshow test			
Chi-squared (*P*-value)	10.6 (0.23)	23.2 (0.010)	6.9 (0.73)
Cox calibration regression			
Intercept (95% CI)	0.036 (−0.029, 0.101)	−0.043 (−0.134, 0.048)	−0.091 (−0.280, 0.098)
Slope (95% CI)	1.001 (0.961, 1.041)	1.047 (0.989, 1.106)	1.014 (0.891, 1.137)
Chi-squared (*P*-value)	2.1 (0.34)	10.8 (0.004)	2.3 (0.32)
Brier's score	0.121	0.115	0.119
Sum-of-squares R-squared	0.240	0.234	0.232
Shapiro's R	0.678	0.688	0.681
Entropy-based R-squared	0.221	0.219	0.211

CI, confidence interval; ROSC, return of spontaneous circulation.

a Measures of model performance are for models using coefficients fitted in the development dataset.
