# Supplementary material for: Development and validation of risk models to predict outcomes following in-hospital cardiac arrest attended by a hospital-based resuscitation team
Source: Resuscitation. 2014 Aug;85(8):993–1000. doi: 10.1016/j.resuscitation.2014.05.004 (PMC4111919; doi:10.1016/j.resuscitation.2014.05.004)
Supplement: Supplementary file 1 [file mmc1.docx]

# Development and validation of risk models to predict outcomes following in-hospital cardiac arrest attended by a hospital-based resuscitation team

Harrison DA, et al. Resuscitation (2014), <http://dx.doi.org/10.1016/j.resuscitation.2014.05.004>

## Appendix A. Supplementary data

### Supplemental Table 1

Application of inclusion and exclusion criteria to development and validation datasets.

|  | Development | | Validation | | External validation | |
| --- | --- | --- | --- | --- | --- | --- |
| Number of hospitals | 122 |  | 143 |  | 21 |  |
| Total number of resuscitation team visits following 2222 calls for cardiac arrest | 18,304 |  | 9694 |  | 1819 |  |
| Excluded (ineligible), *n* (%) | 3580 | (19.6) | 1840 | (19.0) | 124 | (6.8) |
| Pre-hospital arrests | 2666 | (14.6) | 1,283 | (13.2) | 33 | (1.8) |
| Second and subsequent visits to the same patient | 533 | (2.9) | 315 | (3.2) | 58 | (3.2) |
| DNACPR decision documented | 381 | (2.1) | 242 | (2.5) | 33 | (1.8) |
| Eligible patients, *n* | 14,724 |  | 7854 |  | 1695 |  |
| Excluded (missing data), *n* (%) | 36 | (0.2) | 63 | (0.8) | 38 | (2.2) |
| Last known status still in hospital | 7 | (<0.1) | 1 | (<0.1) | 0 | (0) |
| Missing ROSC > 20 min | 5 | (<0.1) | 58 | (0.7) | 37 | (2.2) |
| Missing hospital outcome | 2 | (<0.1) | 0 | (0) | 0 | (0) |
| Missing predictors^a^ | 22 | (0.1) | 4 | (0.1) | 1 | (0.1) |
| Included, *n* (%) | 14,688 | (99.8) | 7791 | (99.2) | 1657 | (97.8) |

DNACPR, do not attempt cardiopulmonary resuscitation; ROSC, return of spontaneous circulation.
^a^ 11 missing age, 5 missing sex, 16 missing prior length of stay, 12 missing reason for admission to/attendance at/visit to hospital, 2 missing location of arrest, 3 missing status at team arrival.

### Supplemental Table 2

Simplification of risk models for return of spontaneous circulation greater than 20 min and hospital survival following in-hospital cardiac arrest attended by a hospital-based resuscitation team.

| Risk model | df | LL | AIC | c index | HL^a^ | *B* | *R* |
| --- | --- | --- | --- | --- | --- | --- | --- |
| **ROSC > 20 min** |  |  |  |  |  |  |  |
| Full model | 37 | −8830 | 17,733 | 0.727 | 64.7 | 0.208 | 0.547 |
| After combining categories | 32 | −8832 | 17,727 | 0.727 | 70.7 | 0.209 | 0.547 |
| Variables removed (*P*-value): |  |  |  |  |  |  |  |
| Deteriorating (0.33) | 31 | −8832 | **17,726** | 0.727 | 72.3 | 0.209 | 0.547 |
| Sex (0.001) | 30 | −8837 | 17,735 | 0.726 | 63.9 | 0.209 | 0.546 |
| Prior LOS (<0.001) | 27 | −8849 | 17,753 | 0.725 | 57.0 | 0.209 | 0.546 |
| Reason (<0.001) | 21 | −8886 | 17,813 | 0.722 | 47.7 | 0.210 | 0.544 |
| Age (<0.001) | 17 | −8951 | 17,935 | 0.711 | - | 0.212 | 0.542 |
| Location (<0.001) | 9 | −9142 | 18,302 | 0.678 | - | 0.219 | 0.533 |
| Rhythm (<0.001) | 2 | −9985 | 19,975 | 0.500 | - | 0.248 | 0.503 |
| After adding interactions^b^ | 46 | −8741 | 17,574 | 0.733 | 24.6 | 0.206 | 0.550 |
|  |  |  |  |  |  |  |  |
| **Hospital survival** |  |  |  |  |  |  |  |
| Full model | 37 | −5768 | 11,610 | 0.804 | 23.1 | 0.123 | 0.674 |
| After combining categories | 32 | −5770 | 11,603 | 0.804 | 21.3 | 0.123 | 0.674 |
| Variables removed (*P*-value): |  |  |  |  |  |  |  |
| Deteriorating (0.76) | 31 | −5770 | 11,601 | 0.804 | 23.0 | 0.123 | 0.674 |
| Sex (0.47) | 30 | −5770 | **11,600** | 0.804 | 18.8 | 0.123 | 0.674 |
| Prior LOS (<0.001) | 27 | −5797 | 11,649 | 0.802 | 14.9 | 0.124 | 0.672 |
| Reason (<0.001) | 21 | −5878 | 11,799 | 0.794 | 25.0 | 0.126 | 0.668 |
| Age (<0.001) | 17 | −6040 | 12,114 | 0.776 | - | 0.130 | 0.660 |
| Location (<0.001) | 9 | −6308 | 12,634 | 0.721 | - | 0.137 | 0.647 |
| Rhythm (<0.001) | 2 | −7188 | 14,379 | 0.500 | - | 0.160 | 0.607 |
| After adding interactions^b^ | 45 | −5677 | 11,444 | 0.811 | 10.6 | 0.121 | 0.678 |

AIC, Akaike Information Criterion; *B*, Brier’s score; df, degrees of freedom; HL, Hosmer-Lemeshow; LL, log-likelihood; LOS, length of stay; *R*, Shapiro’s R; ROSC, return of spontaneous circulation.
^a^ Chi-squared statistic from Hosmer-Lemeshow test based on ten equal sized groups (eight degrees of freedom); ten equal sized groups could not be defined for the models with two or fewer predictors.
^b^ Interactions added to model from stepwise reduction with lowest AIC (indicated in bold text).

### Supplemental Table 3

Final risk model for return of spontaneous circulation greater than 20 minutes following in-hospital cardiac arrest attended by a hospital-based resuscitation team fitted in the full dataset (*N*=22,479).

| Predictor | Patients | ROSC > 20 min, *n* (%) | | Coefficient (95% CI) | |
| --- | --- | --- | --- | --- | --- |
| Age (spline base variables^a^) |  |  |  |  |  |
| age_1_ | - | - |  | −0.0019 | (−0.0066, 0.0027) |
| age_2_ | - | - |  | −0.0118 | (−0.0259, 0.0024) |
| age_3_ | - | - |  | 0.0315 | (−0.1772, 0.2403) |
| age_4_ | - | - |  | −0.1120 | (−0.7397, 0.5157) |
| Sex |  |  |  |  |  |
| Female | 9590 | 4289 | (44.7) | 0 |  |
| Male | 12,889 | 5825 | (45.2) | −0.1167 | (−0.1762, −0.0573) |
| Prior LOS |  |  |  |  |  |
| 0 days | 6276 | 3334 | (53.1) | 0 |  |
| 1 day | 3804 | 1758 | (46.2) | −0.1060 | (−0.2075, −0.0045) |
| 2–7 days | 7136 | 2859 | (40.1) | −0.2350 | (−0.3313, −0.1388) |
| 8 or more days | 5263 | 2163 | (41.1) | −0.1753 | (−0.2785, −0.0722) |
| Reason for attendance |  |  |  |  |  |
| Patient–medical | 18,144 | 7923 | (43.7) | 0 |  |
| Patient–trauma | 854 | 319 | (37.4) | 0.0173 | (−0.1383, 0.1729) |
| Patient–elective surgery | 1461 | 872 | (59.7) | 0.4920 | (0.3671, 0.6170) |
| Patient–emergency surgery | 1706 | 770 | (45.1) | −0.0529 | (−0.1666, 0.0609) |
| Patient–obstetric | 47 | 38 | (80.9) | 1.4674 | (0.7045, 2.2303) |
| Outpatient | 218 | 158 | (72.5) | 0.4281 | (0.0365, 0.8197) |
| Staff or visitor | 49 | 34 | (69.4) | 0.2874 | (−0.3605, 0.9353) |
| Location of arrest |  |  |  |  |  |
| ED | 2357 | 1039 | (44.1) | −0.1061 | (−0.3518, 0.1396) |
| EAU | 1929 | 846 | (43.9) | 0.1853 | (0.0708, 0.2998) |
| Ward, obstetric area, intermediate care area or other inpatient location | 12,912 | 4822 | (37.3) | 0 |  |
| CCU | 2057 | 1263 | (61.4) | 0.9226 | (0.7112, 1.1340) |
| Critical care unit | 1480 | 952 | (64.3) | 0.1712 | (−0.1322, 0.4746) |
| Imaging department or specialist treatment area | 565 | 347 | (61.4) | 0.6062 | (0.0574, 1.1549) |
| Cardiac catheter laboratory | 694 | 507 | (73.1) | 1.0379 | (0.7755, 1.3003) |
| Theatre and recovery | 276 | 193 | (69.9) | 0.3557 | (−0.4972, 1.2085) |
| Clinic or non-clinical area | 160 | 111 | (69.4) | 0.4783 | (0.0423, 0.9143) |
| Presenting rhythm |  |  |  |  |  |
| VF | 2512 | 1821 | (72.5) | 0 |  |
| VT | 1077 | 857 | (79.6) | 0.2960 | (0.1158, 0.4763) |
| Shockable–unknown rhythm | 133 | 75 | (56.4) | −0.4064 | (−0.7771, −0.0357) |
| Asystole | 5454 | 1431 | (26.2) | −2.0035 | (−2.1427, −1.8644) |
| PEA | 11,076 | 4534 | (40.9) | −1.0853 | (−1.2085, −0.9621) |
| Bradycardia | 156 | 110 | (70.5) | −0.0973 | (−0.4974, 0.3029) |
| Non-shockable–unknown rhythm | 492 | 247 | (50.2) | −0.6084 | (−0.8318, −0.3850) |
| Unknown | 1579 | 1039 | (65.8) | −0.0137 | (−0.1767, 0.1493) |
| Interaction between asystole and location of arrest |  |  |  |  |  |
| ED | - | - |  | 0.4118 | (0.0851, 0.7385) |
| EAU, ward, obstetric area, intermediate care area or other inpatient location | - | - |  | 0 |  |
| CCU or cardiac catheter lab | - | - |  | 0.7700 | (0.4761, 1.0639) |
| Critical care unit | - | - |  | 1.4013 | (1.0245, 1.7781) |
| Imaging department or specialist treatment area | - | - |  | 0.1224 | (−0.5778, 0.8225) |
| Theatre and recovery | - | - |  | 1.4432 | (0.3919, 2.4946) |
| Interaction between PEA and location of arrest |  |  |  |  |  |
| ED | - | - |  | 0.0079 | (−0.2537, 0.2695) |
| EAU, ward, obstetric area, intermediate care area or other inpatient location | - | - |  | 0 |  |
| CCU or cardiac catheter lab | - | - |  | −0.8256 | (−1.0672, −0.5840) |
| Critical care unit | - | - |  | 0.4604 | (0.1205, 0.8004) |
| Imaging department or specialist treatment area | - | - |  | −0.0692 | (−0.6623, 0.5239) |
| Theatre and recovery | - | - |  | 0.4798 | (−0.4350, 1.3945) |
| Interaction between other non-shockable/ unknown rhythms and location of arrest |  |  |  |  |  |
| ED | - | - |  | −0.0748 | (−0.4751, 0.3255) |
| EAU, ward, obstetric area, intermediate care area or other inpatient location | - | - |  | 0 |  |
| CCU or cardiac catheter lab | - | - |  | −0.2823 | (−0.7227, 0.1581) |
| Critical care unit | - | - |  | 0.3021 | (−0.2682, 0.8724) |
| Imaging department or specialist treatment area | - | - |  | 0.2937 | (−0.5486, 1.1360) |
| Theatre and recovery | - | - |  | 0.6059 | (−0.7864, 1.9982) |
| Constant | - | - |  | 1.1309 | (0.8574, 1.4043) |
| SD of random effect | - | - |  | 0.3016 | (0.2501, 0.3636) |
| ICC | - | - |  | 0.0269 | (0.0187, 0.0386) |

CCU, coronary care unit; CI, confidence interval; EAU, emergency admissions unit; ED, emergency department; HDU, high dependency unit; ICC, intracluster correlation coefficient; LOS, length of stay; PEA, pulseless electrical activity; PHDU, paediatric high dependency unit; PICU, paediatric intensive care unit; SD, standard deviation; VF, ventricular fibrillation; VT, ventricular tachycardia.

^a^ Spline base variables:
age_1_ = age
age_2_ = [max((age – 42)^3^,0) – 49×max((age – 83)^3^,0)/8 + 41×max((age – 91)^3^,0)/8]/49^2^
age_3_ = [max((age – 67)^3^,0) – 24×max((age – 83)^3^,0)/8 + 16×max((age – 91)^3^,0)/8]/49^2^
age_4_ = [max((age – 76)^3^,0) – 15×max((age – 83)^3^,0)/8 + 7×max((age – 91)^3^,0)/8]/49^2^

### Supplemental Table 4

Final risk model for hospital survival following in-hospital cardiac arrest attended by a hospital-based resuscitation team fitted in the full dataset (*N*=22,479).

| Predictor | Patients | Hospital survival, *n* (%) | | Coefficient (95% CI) | |
| --- | --- | --- | --- | --- | --- |
| Age (spline base variables^a^) |  |  |  |  |  |
| age_1_ | - | - |  | −0.0153 | (−0.0203, −0.0102) |
| age_2_ | - | - |  | −0.0125 | (−0.0295, 0.0044) |
| age_3_ | - | - |  | −0.0133 | (−0.2848, 0.2582) |
| age_4_ | - | - |  | 0.2135 | (−0.6492, 1.0762) |
| Prior LOS |  |  |  |  |  |
| 0 days | 6276 | 1809 | (28.8) | 0 |  |
| 1 day | 3804 | 741 | (19.5) | −0.2339 | (−0.3598, −0.1080) |
| 2–7 days | 7136 | 1061 | (14.9) | −0.4342 | (−0.5535, −0.3149) |
| 8 or more days | 5263 | 752 | (14.3) | −0.4593 | (−0.5897, −0.3289) |
| Reason for attendance |  |  |  |  |  |
| Patient–medical | 18,144 | 3276 | (18.1) | 0 |  |
| Patient–trauma | 854 | 84 | (9.8) | −0.2790 | (−0.5307, 0.0274) |
| Patient–elective surgery | 1461 | 491 | (33.6) | 0.8545 | (0.7136, 0.9955) |
| Patient–emergency surgery | 1706 | 325 | (19.1) | −0.0916 | (−0.2437, 0.0604) |
| Patient–obstetric | 47 | 35 | (74.5) | 2.2917 | (1.5873, 2.9961) |
| Outpatient | 218 | 124 | (56.9) | 0.8693 | (0.4831, 1.2555) |
| Staff or visitor | 49 | 28 | (57.1) | 0.8531 | (0.2298, 1.4765) |
| Location of arrest |  |  |  |  |  |
| ED | 2357 | 457 | (19.4) | 0.2175 | (−0.0267, 0.4618) |
| EAU | 1929 | 291 | (15.1) | 0.1055 | (−0.0534, 0.2644) |
| Ward, obstetric area, intermediate care area or other inpatient location | 12,912 | 1596 | (12.4) | 0 |  |
| CCU | 2057 | 802 | (39.0) | 1.2194 | (1.0355, 1.4033) |
| Critical care unit | 1480 | 408 | (27.6) | −0.0865 | (−0.3600, 0.1869) |
| Imaging department or specialist treatment area | 565 | 188 | (33.3) | 0.2111 | (−0.2413, 0.6634) |
| Cardiac catheter laboratory | 694 | 386 | (55.6) | 1.3527 | (1.1132, 1.5923) |
| Theatre and recovery | 276 | 124 | (44.9) | 0.3853 | (−0.3438, 1.1145) |
| Clinic or non-clinical area | 160 | 83 | (51.9) | 0.6961 | (0.2544, 1.1378) |
| Presenting rhythm |  |  |  |  |  |
| VF | 2512 | 1200 | (47.8) | 0 |  |
| VT | 1077 | 573 | (53.2) | 0.1576 | (−0.0009, 0.3161) |
| Shockable–unknown rhythm | 133 | 36 | (27.1) | −0.4498 | (−0.8724, −0.0273) |
| Asystole | 5454 | 501 | (9.2) | −2.4155 | (−2.6145, −2.2165) |
| PEA | 11,076 | 1316 | (11.9) | −1.5656 | (−1.7048, −1.4264) |
| Bradycardia | 156 | 74 | (47.4) | −0.0027 | (−0.4024, 0.3970) |
| Non-shockable–unknown rhythm | 492 | 110 | (22.4) | −0.6150 | (−0.8771, −0.3529) |
| Unknown | 1579 | 553 | (35.0) | −0.0119 | (−0.1804, 0.1567) |
| Interaction between asystole and location of arrest |  |  |  |  |  |
| ED | - | - |  | 0.3481 | (−0.0661, 0.7623) |
| EAU, ward, obstetric area, intermediate care area or other inpatient location | - | - |  | 0 |  |
| CCU or cardiac catheter lab | - | - |  | 1.3103 | (0.9978, 1.6228) |
| Critical care unit | - | - |  | 1.6794 | (1.2840, 2.0748) |
| Imaging department or specialist treatment area | - | - |  | 0.7625 | (0.0042, 1.5208) |
| Theatre and recovery | - | - |  | 2.0083 | (1.0440, 2.9725) |
| Interaction between PEA and location of arrest |  |  |  |  |  |
| ED | - | - |  | −0.4057 | (−0.7026, 0.1087) |
| EAU, ward, obstetric area, intermediate care area or other inpatient location | - | - |  | 0 |  |
| CCU or cardiac catheter lab | - | - |  | −0.6241 | (−0.8736, −0.3745) |
| Critical care unit | - | - |  | 0.5458 | (0.2082, 0.8833) |
| Imaging department or specialist treatment area | - | - |  | 0.5060 | (−0.0204, 1.0324) |
| Theatre and recovery | - | - |  | 0.8750 | (0.0694, 1.6806) |
| Interaction between other non-shockable/ unknown rhythms and location of arrest |  |  |  |  |  |
| ED | - | - |  | −0.5479 | (−0.9610, −0.1348) |
| EAU, ward, obstetric area, intermediate care area or other inpatient location | - | - |  | 0 |  |
| CCU or cardiac catheter lab | - | - |  | −0.7431 | (−1.1414, 0.3448) |
| Critical care unit | - | - |  | 0.0329 | (−0.5010, 0.5668) |
| Imaging department or specialist treatment area | - | - |  | 0.9988 | (0.2978, 1.6998) |
| Theatre and recovery | - | - |  | 0.1470 | (−0.9878, 1.2817) |
| Constant | - | - |  | 0.8737 | (0.5825, 1.1650) |
| SD of random effect | - | - |  | 0.2850 | (0.2285, 0.3556) |
| ICC | - | - |  | 0.0241 | (0.0156, 0.0370) |

CCU, coronary care unit; CI, confidence interval; EAU, emergency admissions unit; ED, emergency department; HDU, high dependency unit; ICC, intracluster correlation coefficient; LOS, length of stay; PEA, pulseless electrical activity; PHDU, paediatric high dependency unit; PICU, paediatric intensive care unit; SD, standard deviation; VF, ventricular fibrillation; VT, ventricular tachycardia.

^a^ Spline base variables:
age_1_ = age
age_2_ = [max((age – 42)^3^,0) – 49×max((age – 83)^3^,0)/8 + 41×max((age – 91)^3^,0)/8]/49^2^
age_3_ = [max((age – 67)^3^,0) – 24×max((age – 83)^3^,0)/8 + 16×max((age – 91)^3^,0)/8]/49^2^
age_4_ = [max((age – 76)^3^,0) – 15×max((age – 83)^3^,0)/8 + 7×max((age – 91)^3^,0)/8]/49^2^

### Supplemental Fig 1. Participation of hospitals in the National Cardiac Arrest Audit, 1 April 2011 to 31 March 2013.

### Supplemental Fig 2. Numbers of in-hospital cardiac arrests and observed and predicted outcomes by age in five year bands. CI, confidence interval; ROSC, return of spontaneous circulation.
